# HIV合并肺癌的个体化综合治疗

**DOI:** 10.3779/j.issn.1009-3419.2018.04.21

**Published:** 2018-04-20

**Authors:** 琳 王, 言峥 宋

**Affiliations:** 201508 上海，上海市公共卫生临床中心 Department of Cerebral Surgery Public Health Clinical Center Affiliated, Shanghai 201508, China

**Keywords:** 肺肿瘤, 人类免疫缺陷病毒, 个体化, 手术治疗, 化疗, 生存期, Lung neoplasms, Human immunodeficiency virus, Individualized, Surgical treatment, Chemotherapy, Survival rate

## Abstract

**背景与目的:**

观察HIV合并肺癌患者临床特征、临床分期、病理学类型、治疗方案及临床效果，为HIV合并肺癌的个体化综合治疗提供指导。

**方法:**

通过回顾性分析我科收治的53例HIV合并肺癌患者，其中47例非小细胞肺癌（non-small cell lung cancer, NSCLC），6例小细胞肺癌（small cell lung cancer, SCLC），24例接受手术联合化疗，22例接受单纯化疗，7例放弃治疗；其中Ⅰ期-Ⅲ期28例，Ⅳ期25例；28例Ⅰ期-Ⅲ期患者中手术联合化疗24例，2例放弃治疗，2例合并严重慢性阻塞性肺疾病无法耐受手术接受化疗。根据患者治疗前高效抗逆转录病毒治疗（highly active anti-retroviral therapy, HAART）治疗情况，将治疗前接受HAART治疗的视为观察组（*n*=27），未接受HAART治疗患者放入对照组（*n*=19）。对比两组生存率情况，分析患者不同治疗方案的生存率及其独立影响因素。

**结果:**

53例HIV合并肺癌患者中接受治疗的46例，观察组与对照组1年生存率、2年生存率无组间差异；Ⅰ期-Ⅲ期生存期患者1年生存率为76.0%，2年生存率为60.0%。Ⅳ期患者1年生存率为13.6%，2年生存率为0%。24例手术联合化疗患者1年生存率83.3%，2年生存率62.5%；22例接受单纯化疗的患者1年生存率18.0%，2年生存率为0%。

**结论:**

HIV合并肺癌患者经不同的个体化综合治疗可以提高患者生存率，早期手术联合化疗效果显著。

## Abstract (en)

**Background and objective:**

To observe clinical features, clinical stagings, types ofpathology, treatment options and clinical effects of patients suffer from HIV infection combined with lung cancer, and also to provide guidance for individualized comprehensive treatment of HIV combined with lung cancer.

**Methods:**

Through the retrospective analysis of 53 cases of HIV merger of lung cancer patients admitted in our department, 47 cases of non-small cell lung cancer (NSCLC), 6 cases of small cell lung cancer (SCLC), 24 cases accepted surgery combined chemotherapy, 22 patients with simple chemotherapy, 7 cases give up treatment; 28 cases are in stages Ⅰ-Ⅲ, 25 cases are in stage Ⅳ; 24 patients received combined chemotherapy in 28 patients with stages Ⅰ-Ⅲ, 2 cases gave up treatment, 2 cases with severe chronic obstructive pulmonary disease (COPD) could not tolerate chemotherapy plus surgery. According to the situation of patients before highly active anti-retroviral therapy (HAART) treatment, patients who received HAART before treatment were divided into observation group (*n*=27), patients who did not receive HAART were divided into control group (*n*=19). The survival and the independent influencing factors between the two groups were analyzed.

**Results:**

Among the 53 HIV infected cases a toal of 46 patients received treatment among 53 cases of treatment in patients with lung cancer merger of HIV, there are no differences of 1 year survival rate, 2 years survival rate between observation group and control group; patients in Ⅰ-Ⅲ phase 1 year survival rate was 76.0%, 2 years survival rate was 60.0%. Patients in Ⅳ phase 1 year survival rate was 13.6%, 2 years survival rate was 0%. 24 patients with surgery combined chemotherapy 1 year survival rate was 83.3%, 2 years survival rate was 62.5%; 22 cases treated with simple chemotherapy 1 year survival rate was 18.0%, 2 years survival rate was 0%.

**Conclusion:**

HIV merger in patients with lung cancer can improve the patients survival rate after different individualized comprehensive treatment, early surgery with combined chemotherapy has remarkable effect.

人类免疫缺陷病毒（human immunodeficiency virus, HIV）感染的患者发生恶性肿瘤的风险比普通人群明显增高，特别是卡波齐肉瘤和非霍奇金淋巴瘤等HIV相关性肿瘤^[1]^。随着高效抗逆转录病毒治疗（highly active anti-retroviral therapy, HAART）联合应用多种药物治疗HIV感染方案的实施，降低了HIV感染的发病率和死亡率，包括由艾滋病诱发的肿瘤的发生率。但是，其他非艾滋病相关的肿瘤的发生率却在上升^[2]^。肺癌是最常见的非艾滋病相关的肿瘤之一，由于肺癌的发病率与年龄有关，在有效的联合抗逆转录病毒疗法的情况下，随着人类免疫缺陷病毒感染者寿命的延长，已成为高收入国家人类免疫缺陷病毒感染者的主要死亡原因^[3]^。但关于HIV合并肺癌患者针对性治疗及生存质量的研究报道极少，近年研究重点集中在两者间的相互关系及危险因素、预后研究^[4, 5]^，是否对此类患者采取积极的综合治疗成为业界争论的焦点。本研究通过对53例HIV合并肺癌患者的临床特征、临床分期、病理学类型、治疗方案及临床效果等进行前瞻性研究，为此类特殊人群研究积累重要的临床材料。

## 1 资料与方法

### 1.1 一般资料

筛选我科自2011年10月-2017年12月收治的HIV合并肺癌患者53例，其中男性47例，女性6例，平均年龄56.90岁。

#### 1.1.1 入组标准

入组标准：①年龄不拘；②性别不拘；③符合1993年美国疾病控制与预防中心（Centers for Disease Control and Prevention, CDC）制订的成人HIV/艾滋病（acquired immuno deficiency syndrome, AIDS）诊断标准。经当地疾控中心实验室进行Western blot（WB）确证试验，确认抗HIV-1抗体阳性；④符合国际肺癌研究会（International Association for the Study of Lung Cancer, IASLC）第八版肺癌TNM分期解读^[6]^；⑤经细胞学或病理组织确诊肺癌。

#### 1.1.2 排除标准

① 排除其他恶性肿瘤患者；②排除患有严重的心、脑、肾、血管等疾病患者；③排除各种严重精神疾患患者。

#### 1.1.3 伦理学考量

① 征求患者及其直系家属同意并签署知情同意书；②充分保障患者的医疗治疗和安全。③保护患者的隐私权，包括患者的信息及诊疗记录。④试验中遵循《渥太华工作关于临床试验注册》的声明。

#### 1.1.4 患者临床特征、肿瘤分布、病理学类型及临床分期

53例HIV合并肺癌患者中男47例，女性6例，平均年龄为56.90岁。体检发现22例，咳嗽、咳痰伴胸痛者18例，痰中带血者8例，发热、乏力、胸闷者5例。吸烟史27例，吸烟量平均在25.0支/天，吸烟时间平均30.1年。既往或现伴发肺部感染者11例，全部为结核感染。37例周围型肺癌，16例中央型肺癌。病理学类型及临床分期详见表 1。

**1:** HIV合并肺癌的病理学类型及临床分期 Pathological types and clinical stages of lung cancer patients infected with HIV

Pathological type	Stage Ⅰ	Stage Ⅱ	Stage Ⅲ	Stage Ⅳ
SCC (*n*=11)	5	1	1	4
AD (*n*=31)	12	3	2	14
LCC (*n*=3)	1	1	0	1
NC (*n*=1)	0	0	0	1
AC (*n*=2)	1	1	0	0
SCLC (*n*=5)	0	0	0	5
SCC: squamous cell carcinoma; AD: adenocarcinoma; LCC: large cell carcinoma; NC: neuroendocrine carcinoma; AC: atypical carcinoid; SCLC: small cell lung cancer.				

### 1.2 治疗者的分组

将接受治疗者46例患者分为两组，治疗前接受HAART治疗患者放入观察组（*n*=27），未接受HAART治疗患者放入对照组（*n*=19）。

#### 1.2.1 治疗情况

##### 1.2.1.1 HAART治疗情况

27例患者肺癌明确前接受HAART治疗，最长疗程168个月，最短疗程2个月，平均46.9个月，19例治疗前未接受HAART治疗。HAART治疗方案：两种核苷类抗逆转录酶抑制剂联合一种非核苷类抗逆转录酶抑制剂（2NRTIS+1NNRTI），即包括国家卫生部提供的HIV免费治疗药物：齐多夫定（AZT，英国葛兰素史克），拉米夫定（3TC，英国葛兰素史克），司他夫定（D4T，英国葛兰素史克），奈韦拉平（NVP，英国葛兰素史克），依非韦伦（EFV，英国葛兰素史克）。治疗方案为：AZT+3TC+EFV（或NVP）一线方案。

治疗前CD4^+^计算水平分布在3个/mm^3^-641个/mm^3^，平均CD4^+^ T淋巴细胞计数：342.92个/mm^3^。EFV：治疗前HIV病毒载量：最高2.01E+05 copy/mL，最低 < 40 copy/mL（低于检测下限）。其中低于40 copy/mL的20例。

##### 1.2.1.2 手术联合化疗治疗情况

24例患者接受手术联合化疗，手术均行常规肺叶切除+纵隔淋巴结及肺内淋巴结清扫。15例腺癌、2例大细胞肺癌及2例不典型类癌术后接受培美曲塞钠+顺铂方案化疗，5例鳞状细胞癌患者术后接受多西他塞+奈达铂方案化疗。

##### 1.2.1.3 化疗治疗情况

22例Ⅳ期患者接受化疗，化疗方案：含铂双药联合化疗：培美曲塞（500 mg/m^2^，d1；美国礼来公司）+顺铂（75 mg/m^2^，d1；美国礼来公司）；多西他赛+奈达铂；吉西他滨+奈达铂。

### 1.3 观察指标

① 观察两组不同临床分期患者治疗后1年、2年生存率情况。②评估两组患者化疗治疗后不良反应进行评估。③分析治疗前选择HAART的时间、HIV病毒载量、CD4^+^ T淋巴细胞计数指标对两组患者生存期因素分析。

### 1.4 统计学处理

所有研究数据选用统计软件包SPSS 20.0进行统计分析，计数资料采用率（%）表示，组间比较采用卡方检验；计量资料采用均数±标准差（Mean±SD）表示，组间比较采用*t*检验，以*P* < 0.05为差异有统计学意义。

## 2 结果

### 2.1 两组治疗后生存率指标比较

两组患者均包括手术联合化疗及单纯化疗方案。两组数据显示，临床各期患者1年生存率和2年生存率差异无统计学意义（*P* > 0.05）。详见表 2。

**2:** 两组1年生存率、2年生存率指标比较 Comparison of 1-year survival rate and 2-year survival rate between two groups

Group	HAART treated（*n*=27）			HAART untreated（*n*=19）		*χ*^2^	*P* value
	1-survival rate	2-survival rate		1-survival rate	2-survival rate		
Stage Ⅰ	75%	58.3%		83.3%	66.7%	0.023, 8	> 0.05
Stage Ⅱ	66.7%	33.3%		100%	100%	0.044, 4	> 0.05
Stage Ⅲ	100%	100%		0%	0%	0.074, 1	> 0.05
Stage Ⅳ	27.2%	0%		0%	0%	1.818, 2	> 0.05
	55.5%	33.3%		42.1%	36.8%		> 0.05

### 2.2 两组化疗前后骨髓抑制指标比较

HAART联合化疗组骨髓抑制发生率为85.2%，1度-4度骨髓抑制病例分别为：4例、7例、8例、5例，构成比为14.8%、25.9%、29.6%、18.5%；3例4度骨髓抑制患者给予输血等对症处理无死亡病例。单纯化疗组骨髓抑制发生率为：33.33%，1度-4度骨髓抑制病例分别为：2例、3例、2例、0例，构成比为10.5%、15.7%、10.5%、0%（表 3）。

**3:** 两组化疗前后骨髓抑制指标比较 Comparison of bone marrow suppression indexes between two groups before and after chemotherapy

Group	Haartcombined chemotherapy group（*n*=27）			Chemotherapy group（*n*=19）		*t* value	*P* value
	Pre- chemotherapeutic	Post- chemotherapeutic		Pre- chemotherapeutic	Post- chemotherapeutic		
Hemoglobin（g/L）	118.6±10.12	95.4±27.10		124.63±9.67	116.03±17.87	2.026	< 0.05
Leukocyte（10^9^/L）	5.04±1.41	3.42±1.49		5.94±1.57	4.95±1.63	2.034	< 0.05
Granulocytes（10^9^/L）	3.13±1.15	1.84±0.95		3.96±1.45	3.16±1.15	2.163	< 0.05
Platelet（10^9^/L）	209.9±62.45	186.23±96.19		248.12±64.34	221.09±93.67	2.018	< 0.05

### 2.3 两组生存率单因素影响分析

通过对两组生存期发现治疗前是否HAART治疗，生存期不统计学，该类特殊人群的生存期与治疗前CD^+^计数及是否吸烟有显著差异（图 1）。

**1:**
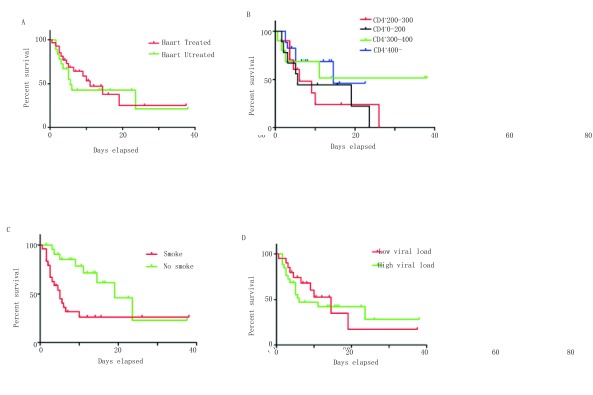
两组生存单因素分析 Single-factor analysis of survival rate in two groups

## 3 讨论

HIV病毒是一种感染人类免疫系统细胞的慢病毒（lentivirus），进入机体后，破坏T淋巴细胞，使机体内CD4^+^ T淋巴细胞数量不断减少，HIV血浆病毒载量明显升高，容易发生各种机会性感染及肿瘤。随着HAART治疗HIV以来，HIV感染患者生存期延长，导致艾滋病非相关性肿瘤发生率呈现上升趋势，特别是HIV感染合并肺癌。Hasse指出，HIV感染者肺癌的发病率是健康人的2.5倍^[7]^。目前比较认同的HIV合并肺癌的风险因素包括：吸烟、HIV病毒本身、高级免疫抑制的存在及慢性肺部炎症^[8-10]^。由于艾滋病毒本身可能会激活原癌基因，导致细胞周期调节的改变，或导致肿瘤基因发生改变。另一方面，HIV相关的免疫抑制可能导致慢性免疫激活、炎症和免疫系统功能紊乱，这些都可能增加罹患癌症的风险。但是，CD4^+^ T细胞的数量或者免疫抑制的持续时间与肺癌发生率的关系尚不清楚^[11-13]^。

HIV合并肺癌在年轻人群中更为常见，比普通人患肺癌平均提前10年-15年^[4, 14-20]^。而且，绝大多数HIV合并肺癌患者都被诊断为晚期，一半以上的患者为Ⅲ期或Ⅳ期^[2, 21-23]^。腺癌是最为普遍的类型，其次是其他非小细胞癌、大细胞癌及小细胞肺癌。通常，HIV合并肺癌的患者预后和整体生存率较差^[1, 2, 15, 20, 24, 25]^。

目前，对于HIV合并肺癌没有明确的治疗指南，就笔者经验而言，如果一个HIV阳性患者被诊断为肺癌，而且已经开始接受HAART治疗，那么就应该根据患者一般情况、肿瘤分型及分期选择手术或采用合适的抗肿瘤药物来增加治疗机会。另一方面，如果一个患者同时被诊断为HIV和肺癌，首先应该选择肺癌的治疗，然后再考虑抗病毒治疗。

### 3.1 外科治疗

HIV合并肺癌是否应采用积极的根治性手术治疗及何时手术时机最佳尚存在争议，主要有以下三方面困惑。首先是由于患者免疫系统的破坏，手术的危险性和并发症是否会明显增加以及手术并发症与患者术前哪些指标有相关性，目前国内外都无明确的定论。笔者通过对该类患者手术后观察发现，术后HIV合并肺癌胸腔渗出明显多于普通患者，且与HIV病毒载量呈正相关，HIV病毒载量与手术后肺部感染发生率呈负相关^[22]^。表明HIV感染问题不是手术禁忌症，绝大多数HIV感染患者除了肺部感染的发生率增加外，手术并发症的危险性并无增加。再次是HIV合并NSCLC患者手术指征的把握，已有的经验显示，HIV病毒载量高于30, 000 copy/mL且CD4细胞计数低于200的HIV感染者与其他HIV感染者相比，出现术后并发症的人数更多。术后并发症主要包括肺部感染、住院时间延长以及细菌感染等^[25-29]^。笔者认为，只要术前在患者身体条件耐受手术的情况下，术前注意预防患者肺部机会性感染，在积极的HAART下，应遵循未感染HIV非小细胞肺癌的手术指征。最后是HIV合并NSCLC患者术前HAART时间及术前HIV各项指标不同是否能够获得相似的生存期。有证据^[30]^表明，HAART期间，HIV合并NSCLC患者经积极治疗后可以获得与未感染HIV患者类似的生存期。

### 3.2 化疗

HIV感染合并肺癌的患者建议采用HAART和化疗联合治疗。但由于HART药物与抗肿瘤药物之间可能产生过多的药物毒性或降低药效及药物的叠加效应，应充分考虑抗肿瘤药物与抗病毒药物的相互作用。建议肿瘤专家和HIV专家联合制定出合适的抗肿瘤和HART疗法，以最大限度地增加疗效降低毒性。

### 3.3 HIV合并肺癌的诊断及治疗上存在问题和展望

HIV合并肺癌的诊断存在的问题：研究显示：5%-15%的HIV感染患者于发现肺癌时无症状。故HIV感染早期肺癌临床表现不明显，其鉴别和诊断存在一定的难度，根据HIV感染患者生活上是否有一般引起肺癌的因素如吸烟，电离辐射，诱发肺癌的饮食等致病因素存在，建议低剂量CT扫描进行筛查，尽可能及早发现早期患有肺癌的HIV感染患者。

更多的肿瘤临床试验应用于HIV合并肺癌：在肿瘤临床试验中，HIV感染的患者通常都被排除在外，尤其在使用新的药物组合来治疗肺癌的临床试验中，排除的因素包括：HART和细胞抑制剂或分子靶标的联合用药存在潜在风险；感染者免疫系统受到严重损伤；目前所使用的HARRT治疗药物是安全的。而且，在HART后，HIV生存率提高，影响生存率的主要因素是肺癌本身，因为肺癌是HIV感染者致死的一个主要原因。因此，在HIV感染可控制的条件下，将HIV感染合并肺癌患者放入试验组中是可行的。近年来，PD-1抑制性细胞受体引起了HIV研究的注意。PD-1在CD4、CD8、NK T细胞、B细胞和单核细胞中表达，在急性和慢性的HIV感染中都存在。在HIV感染过程中，PD-1下调CD4、CD8和B细胞，从而导致免疫损伤。前期的研究表明，PD-1阻断可调节体内病毒特性CD8细胞机能恢复，同样受损伤的CD4细胞也可能恢复，B细胞可以重新产生病毒特异性抗体。近期有报道阐述了PD-1抑制剂在肺癌中的功效。PD-L1和P-DL2，在肿瘤细胞和肿瘤浸润免疫细胞中表达。当PD-L1高表达时，PD-1抗体非常有效。此外，与标准的二线治疗相比，PD-1抗体作用显著，而且明显毒性偏低。希望PD1/PDL1能够尽早应用于HIV合并肺癌的临床试验。

综上所述，HIV合并肺癌患者积极实行个体化综合治疗，在HAART同时，可以提高患者的生存率。
